# Between difficulty and manageability: beliefs about the learnability of German in Turkish-language YouTube comments

**DOI:** 10.3389/fpsyg.2026.1926209

**Published:** 2026-10-08

**Authors:** Ebru Üstün

**Affiliations:** Department of German Language Teaching, Faculty of Education, Nevşehir Hacı Bektaş Veli University, Nevşehir, Türkiye

**Keywords:** cognitive appraisal, generative AI, learner beliefs, learning motivation, perceived language difficulty, self-efficacy, user-generated data

## Abstract

Whether a foreign language is experienced as difficult is less an objective property of the language system than a subjective belief, linked to language attitudes and to beliefs about one’s own capacity to cope, that may influence motivation and learning behavior. How the difficulty of German is interpreted in Turkish-speaking learner discourse has so far received little attention. From a learning-psychology perspective, the present study reconstructs the beliefs, attitudes, and coping expectations articulated in user-generated data, asking which features of the German language commenters identify as difficult or manageable, which overarching belief and attitude patterns emerge, and how commenters negotiate the relationship between perceived difficulty and confidence in coping. The data comprised 3,884 Turkish-language comments on four YouTube videos about the German language, collected in June 2026 using YouTube Data Tools; of these, 2,085 were classified as thematically relevant and analyzed by means of qualitative thematic analysis following Braun and Clarke. Because commenters cannot be verified as learners, the comments are treated as the discourse of a self-selected online community engaging with the learning of German, in which learners and learning-related experience predominate. A category system with eight main categories was developed. The results show a clear predominance of comparative and process-related categories over categories addressing individual phenomena: difficulty is negotiated primarily through cross-linguistic comparison and multilingualism (51.9%) and through learning- and acquisition-related aspects (50.2%), and only secondarily through individual phenomena. The characteristic pattern that emerged was the coexistence of a high perception of difficulty with pronounced confidence in coping (“difficult but learnable”). Since self-efficacy was not measured, this pattern is interpreted as a belief pattern consistent with, but not evidence of, an interplay of perceived difficulty and self-efficacy. In a small number of the most recent comments, some commenters additionally invoked AI tools as a coping resource, an exploratory observation that points to a possible direction for future research. The study demonstrates the value of user-generated digital data for researching beliefs about language difficulty and its manageability.

## 1 Introduction

Whether a foreign language is experienced as difficult is less an objective property of the language system than a subjective belief held by learners. In the psychology of language learning, such learner beliefs are regarded as significant predictors of motivation, willingness to invest effort, and learning behavior (Horwitz, 1988). Closely related are language attitudes as well as self-efficacy beliefs, that is, the conviction that one can manage the demands of a language through one’s own actions (Çobanoğulları, 2022). German is widely regarded as difficult among learners; from a psychological perspective, this attribution is a perception that may become effective within the learning process, potentially shaping expectations, emotions, and persistence. For Turkish-speaking learners, this question arises with particular acuity, as profound structural differences exist between agglutinating, genderless Turkish and inflecting German with its rich system of articles and cases, differences that may intensify perceived difficulty.

How learners experience, interpret, and articulate this difficulty has so far received little systematic investigation. With the rise of social media, however, a space has emerged in which learners articulate their beliefs, attitudes, and emotional evaluations spontaneously and largely without censorship. The comment sections beneath language-learning videos on YouTube constitute one such space: here, commenters spontaneously address the difficulty of German, compare it with other languages, recount their own learning experiences, and negotiate whether and how the language can be mastered. Such user-generated data offer an ecologically valid access to language-related beliefs and attitudes, one that is less subject to the distortions of direct surveys, such as socially desirable responding (Benson, 2015), although they carry limitations of their own, which are discussed in Section 2.6.

The present study connects these two starting points from a learning-psychology perspective. It analyzes Turkish-language YouTube comments on the difficulty of German by means of qualitative thematic analysis, in order to reconstruct which beliefs, attitudes, and self-efficacy perceptions underlie the statements about the difficulty of German, and how learners negotiate the tension between perceived difficulty and confidence in coping. The primary theoretical lens of the study is the concept of learner beliefs (Horwitz, 1988), within which self-efficacy (Bandura, 1997) is treated as the central belief construct. Cognitive appraisal theory (Lazarus and Folkman, 1984) serves as the explanatory mechanism that links these beliefs to the perception of a language as difficult or manageable, while motivational theory (Dörnyei, 2009) and language attitude research (Garrett, 2010) provide complementary perspectives on the consequences and the social embedding of such beliefs. The study was undertaken because the relevant research strands have so far remained separate: the psychology of learner beliefs has rarely examined how such beliefs are articulated outside institutional settings, the contrastive literature on the Turkish-German language pair describes structural contrasts without examining how they are experienced, and research on YouTube discourse has not yet been applied to Turkish-speaking learners of German. The contribution of the study is accordingly twofold. Empirically, it provides a qualitative reconstruction, not previously available, of how Turkish-speaking commenters negotiate the difficulty of German in authentic, user-generated discourse. Theoretically, it identifies a belief pattern in which the acknowledgment of difficulty coexists with confidence in coping, and it shows how this pattern can be interpreted through the interplay of belief and appraisal. The following section brings together the research strands relevant to this question and specifies the research gap that the study addresses.

## 2 Theoretical framework and state of research

The following review is organized around a single primary lens, learner beliefs, and three complementary perspectives whose functions are distinct. Learner beliefs, in the sense of Horwitz (1988) form the primary lens because the study aims to reconstruct what commenters believe about the difficulty and learnability of German; self-efficacy (Bandura, 1997) is treated as the belief construct most directly relevant to this question, since it concerns the belief in one’s own capacity to cope with the language. Cognitive appraisal theory (Lazarus and Folkman, 1984) is not used as a second, competing framework but as the mechanism that explains how such beliefs translate into the perception of a language as a threat or as a manageable challenge; it therefore provides the interpretive key for the central pattern of the study. The L2 Motivational Self System (Dörnyei, 2009) is drawn on to situate the motivational consequences of these beliefs, and language attitude research (Garrett, 2010) to account for their social and evaluative dimension. Contrastive linguistics (Section 2.4) and research on the Turkish GFL context (Section 2.5) do not contribute further psychological constructs but describe the structural and institutional background against which the beliefs arise. This ordering is maintained in the discussion, where the findings are interpreted primarily through the lens of beliefs and self-efficacy and explained through appraisal, with motivation and attitudes as complementary perspectives.

### 2.1 Perceived language difficulty and learner metaphors

The subjective perception of language difficulty is regarded as a decisive factor in the learning process and can be understood psychologically as an appraisal that influences learners’ emotions, expectations, and behavior. In direct comparisons of different foreign languages, learners’ attitudes and difficulty judgments vary (Kovac and Mrsic, 2017). To uncover the often implicit attitudes and beliefs underlying such judgments, metaphor analysis has proven a productive approach (Krüsemann and Graham, 2024). In the British context, learners frequently associate the German language metaphorically with difficulty and toil, or conceive of it as unchangeably difficult (Krüsemann and Graham, 2024). A similar picture emerges among learners of English in the Turkish context, where the learning process is metaphorically experienced as a continuous, strenuous task demanding considerable willpower (Baş and Bal Gezegin, 2015). Theoretically, this appraisal can be linked to the stress theory of Lazarus and Folkman (1984). Applying their concept of cognitive appraisal to language learning, beliefs function as a formative perceptual lens that determines whether a demand is interpreted as a threat or as a manageable challenge; a situation is appraised as a challenge when the person trusts in their own coping resources (Lazarus and Folkman, 1984).

Across these studies, there is agreement that foreign language learning is frequently conceptualized as an arduous journey or a difficult task (Baş and Bal Gezegin, 2015; Krüsemann and Graham, 2024). Of particular relevance for the psychology of learning is the distinction drawn by Krüsemann and Graham (2024): when learners use dynamic metaphors that portray the learning process as developable, the perceived difficulty does not necessarily lead to demotivation but can instead be interpreted as a manageable challenge, a pattern closely linked to high self-efficacy beliefs. While metaphor studies on English in the Turkish context (Baş and Bal Gezegin, 2015) and on German in the British context (Krüsemann and Graham, 2024) exist, an investigation of the beliefs of Turkish-speaking learners directed specifically at the German language is lacking.

### 2.2 Language attitudes in the Turkish context

In the Turkish educational context, attitudes toward the German language are predominantly instrumentally positive, drawing on motives such as tourism, academic study, and economic relations with the European Union (Sağın Şimşek et al., 2007). Prospective German teachers in Turkey likewise display generally positive attitudes, although these tend to decline slightly over the course of their studies (Alyaz, 2011). Attitudes are also strongly shaped by the societal and political presence of a language (Krüsemann and Graham, 2024). In the sociolinguistic context of the Turkish diaspora in Germany, factors such as level of education, income, and generational membership prove decisive for how positive attitudes toward the mother tongue and toward German turn out to be (Sakin, 2018). From a psychological perspective, language attitudes are not directly observable, multidimensional constructs composed of cognitive, affective, and behavioral components that must be inferred indirectly from verbal statements and reactions (Garrett, 2010).

The sources agree that, in the Turkish context, instrumental and extrinsic motives are the most important drivers of positive language attitudes toward German (Sağın Şimşek et al., 2007). What remains open in this literature, however, is how such a rather rational, utility-oriented attitude changes when learners encounter concrete learning barriers of the German language in informal exchange.

### 2.3 Learner beliefs and self-efficacy

Beliefs about language learning govern learners’ approach and satisfaction (Horwitz, 1988). The Beliefs About Language Learning Inventory (BALLI) was created as an instrument that systematically captures such beliefs and identifies, among other dimensions, the perceived difficulty of language learning as a distinct dimension (Horwitz, 1988). Turkish learners hold specific beliefs that frequently center on vocabulary and grammar learning (Ariogul et al., 2009; Ataş, 2012). The belief in one’s own capability, self-efficacy, is regarded as a strong predictor of learning success; for Turkish learners of German, a measurement instrument has been developed that captures self-efficacy across three dimensions: language learning performance, linguistic skills, and learning confidence (Çobanoğulları, 2022). Fundamental to this construct is Bandura's (1997) concept of perceived self-efficacy, that is, a person’s belief in their capability to organize and execute the actions required to attain a goal. Bandura (1997) conceives of self-efficacy not as a global personality trait but as a domain- and task-specific belief system; what is relevant here, accordingly, is the specific self-efficacy with respect to managing foreign language acquisition. From a motivational-psychology perspective, Dörnyei's (2009) L2 Motivational Self System complements this relationship: motivation arises from the endeavor to reduce the discrepancy between the actual self and an aspired ideal second-language self, whereby this vision unfolds its motivating force only when it is perceived as plausible and attainable through one’s own effort (Dörnyei, 2009).

More recent approaches emphasize that motivation and beliefs are closely embedded in a multilingual identity, as described by the L2 Motivational Self System (Boo et al., 2015), and that this identity is shaped by evaluations, emotions, and experiences (Forbes et al., 2024). During transitional phases of the educational system, such beliefs can fluctuate considerably (Courtney, 2017). The literature converges on the view that positive learner beliefs and high self-efficacy beliefs are the most important internal factors for sustaining motivation, particularly when a language is perceived as difficult (Çobanoğulları, 2022; Krüsemann and Graham, 2024). What is lacking, however, are qualitative studies on how the self-efficacy of Turkish learners behaves in direct contact with the structural asymmetries of German and how it is articulated on social media.

A strand of recent research that connects learner beliefs directly to self-efficacy and motivation is the work on language mindsets, that is, beliefs about whether the ability to learn a language is fixed or can be develop through effort (Lou and Noels, 2017). Learners who hold a growth mindset tend to interpret difficulty as surmountable, persists in the face of setbacks, and show higher self-efficacy and engagement; growth mindsets have been found to predict self-efficacy, which in turn mediates their effect on engagement and perceived proficiency (Kim, 2024; see also Bai and Wang, 2023). Two studies further link mindsets to Dörnyei’s (2009) motivational framework: growth mindsets predicted engagement via the ideal L2 self (Sadoughi et al., 2023), and, in a sample of 403 L2 learners, growth mindsets were related to the ideal L2 self through perseverance of effort (Kırmızı et al., 2023). For the present study, this strand is relevant because the belief that a difficult language remains learnable through effort is, in substance, a growth-oriented belief about language learning; whether and how such beliefs are articulated spontaneously, outside instruments and classrooms, is precisely what the comment data allow to be examined.

A further factor that may increasingly shape these beliefs is the growing availability of generative artificial intelligence (AI). Recent studies suggest that the use of AI tools can be associated with learners’ self-efficacy, motivation, and reduced language anxiety (Kittredge et al., 2025; Li et al., 2025; Guo and Zheng, 2026), which positions such tools as a potential coping resource when learners appraise the difficulty of a language. Beliefs about one’s capability to use AI, that is, AI self-efficacy, are themselves a domain-specific efficacy belief in Bandura’s sense; in a sample of 535 pre-service Teachers in Türkiye, self-regulated engagement with AI built such beliefs, but its effect was strongly conditional on critical thinking disposition (Başaran, 2026). This indicates that access to AI does not translate into efficacy beliefs automatically. Whether and how Turkish-speaking learners of German draw on AI in this way, however, has not yet been examined and is considered here only as an exploratory, secondary aspect.

### 2.4 Contrastive linguistics of the Turkish-German language pair

In order to situate the perceived difficulty of German psychologically, the structural background against which it arises must be taken into account. Turkish and German exhibit fundamental typological divergences. At the morphological level, Turkish is an agglutinating, suffixing language in which meaning-bearing units are appended as unambiguous endings to an invariable word stem, governed by the strict phonological rules of major and minor vowel harmony; German, by contrast, is strongly inflecting and uses both internal inflection through ablaut and external inflection (Ceylan, 2018). Particularly consequential for Turkish learners is that Turkish entirely lacks grammatical gender as well as a system of definite articles, which leads to substantial transfer errors in German (Ceylan, 2018; cf. Balcı, 2009).

At the syntactic level, too, the two languages diverge markedly. German word order is strongly bound to the position of the finite verb (second position in main clauses, final position in subordinate clauses), whereas Turkish is a strictly head-final, left-branching language with the canonical sequence subject–object–predicate that embeds subordinate clauses before the head word (Köppel, 2017). These are described in the contrastive literature as among the most consequential structural contrasts for Turkish learners of German.

The contrasts extend to orthography, phonetics, and word formation: Turkish possesses sounds without a German equivalent, the two writing systems assign different values to comparable graphemes, and while German relies heavily on prefixes, suffixes, and parasynthesis, Turkish forms new words almost exclusively through its rich inventory of suffixes (Kalkavan, 2012; Ceylan, 2018).

The linguistic sources complement one another without contradiction at the morphological (Ceylan, 2018), syntactic (Köppel, 2017), and orthographic-phonetic levels (Kalkavan, 2012), and they consistently document the enormous typological distance between the two languages. These asymmetries, the absent gender, the SOV versus V2 order, and the left- versus right-branching syntax, are regarded in the contrastive literature as the principal systemic sources of interference errors. From a learning-psychology perspective, they describe the structural distance between the two languages; whether and to what extent this distance is experienced as difficulty is a separate, empirical question. The contrastive literature, however, describes the structural contrasts and the error types associated with them; it does not investigate how Turkish-speaking learners subjectively experience these contrasts, which beliefs and coping expectations they associate with them, and how this metalinguistic discourse is articulated in digital spaces. It is precisely this subjective, psychological perspective that the present study opens up.

### 2.5 German as a foreign language in the Turkish context

Owing to historical ties and the close personal connections brought about by migration, the German language enjoys a special status in Turkey and is classically taught as the second foreign language after English; at a large proportion of Anatolian high schools (a selective, academically oriented secondary school type in Turkey), German is the second foreign language (Karbi, 2020). This context shapes the conditions under which learners’ beliefs and attitudes toward German develop: German is frequently learned on the basis of existing knowledge of English and in direct comparison with the typologically close English, which substantially influences perceived difficulty and coping expectations.

Closely linked to these framing conditions is the multilingualism of the learners. As a pedagogical approach, tertiary language didactics (German after English) is therefore advocated, in which English is systematically used as a bridge language, for instance when comparing vocabulary and sentence structure (İşigüzel, 2025). The digital dimension of this context is not new: technology-supported approaches, including computer-assisted German teaching, have been part of German teacher education at Turkish education faculties for more than a decade (Başaran, 2015) so that today’s learners encounter German in a setting in which digital media are an established part of instruction an of informal learning alike. The concrete role of multilingualism in German instruction, however, remains ambivalent: while multilingualism is welcomed programmatically, its actual effect is in many cases unclear (Janík, 2026).

Research on the Turkish German as a foreign language (GFL) context thus opens up the institutional and multilingual framing conditions of German learning in Turkey (Karbi, 2020; İşigüzel, 2025; Ünal, 2025). Within this field, however, one perspective remains open: how learners themselves perceive the difficulty of the German language, which beliefs and attitudes they associate with it, and how they articulate this in informal, digital spaces beyond institutional instruction, has so far not been systematically investigated. It is precisely at this learner-side, learning-psychology perspective that the present study begins.

### 2.6 YouTube comments as an object of research

YouTube functions not only as a video platform but, in its asynchronous comment sections, constitutes an extensive textual space of computer-mediated communication (Benson, 2015). These comments can be understood as interactive discourses and are particularly well suited to qualitative analysis (Benson, 2015). By means of thematic analysis following Braun and Clarke (2006), collective interpretations and discourses can be elicited from such comments (Liew and Hassan, 2021). For language learning research, corpora of YouTube comments offer uncensored insights into authentic learner and youth language (Cotgrove, 2023; Ivković, 2013). Especially revealing are the comment sections beneath language-learning videos, in which lively metalinguistic discourses about the difficulty, the sound, and the status of languages are conducted (Chowchong, 2021). Since language attitudes must be inferred indirectly from verbal statements (Garrett, 2010), such comments constitute precisely those linguistic data that allow inferences about the underlying, not directly observable beliefs and attitudes.

At the same time, the specific character of comment data imposes limits on what can be inferred from them. First, comments are not neutral reports of belief but situated contributions to a public exchange: they respond to the framing of a particular video, to preceding comments, and to an anonymous audience, so that positions may be sharpened, exaggerated, or performed for effect, and irony, humor and provocation are part of the register. Second, commenters remain largely anonymous; the data provide no verified information on age, proficiency, learning history, or whether a commenter is in fact learning German, so that statements can be attributed to a discourse community but not to a defined population. Third, which videos and comments become visible is co-shaped by platform logics of search ranking and recommendation (Rieder et al., 2018), and comment sections are dynamic and can be edited or deleted, so that any corpus is a time-bound snapshot. Fourth, comment data document expressed evaluations, not learning behavior or actual proficiency, and the beliefs of those who do not comment remain invisible. These characteristics do not invalidate comment data as a source for the reconstruction of beliefs and attitudes; they do, however, define the appropriate level of inference, namely the reconstruction of how difficulty is talked about within a self-selected online discourse rather than the measurement of beliefs in a population. The methodological consequences of these limitations for the present study are addressed in Section 3.6.

This methodological suitability, together with its limits, is well documented, but the application of comment data to the question pursued here remains open: while studies on metalinguistic discourses (Chowchong, 2021) and on identity construction in YouTube comments (Liew and Hassan, 2021) exist, the qualitative analysis of Turkish-language comments in which learners articulate their beliefs and attitudes regarding the difficulty of German has not yet been undertaken.

### 2.7 Research gap and research questions

The research strands reviewed are each well documented in their own right. Research on learner beliefs and language attitudes provides established psychological constructs in the form of beliefs, self-efficacy, and language attitudes (Horwitz, 1988; Bandura, 1997; Garrett, 2010; Çobanoğulları, 2022), which are complemented by appraisal and motivation models (Lazarus and Folkman, 1984; Dörnyei, 2009). Contrastive linguistics describes the structural hurdles of German for Turkish learners with precision (Ceylan, 2018; Köppel, 2017; Kalkavan, 2012), research on the Turkish GFL context opens up its institutional and multilingual framing conditions (Karbi, 2020; İşigüzel, 2025), and digital discourse research identifies YouTube comments as a valid data source for language-related attitudes (Benson, 2015; Chowchong, 2021). None of the available works, however, brings these strands together. What is lacking is an empirical study that reconstructs, from a learning-psychology perspective, which beliefs, attitudes, and coping expectations Turkish-speaking learners associate with the difficulty of German, and that draws for this purpose on the authentic, informal discourse in YouTube comments.

The present study addresses this intersection. From a learning-psychology perspective, it examines which beliefs, attitudes, and coping expectations are expressed with respect to the difficulty of the German language in Turkish-language YouTube comments. From this, three research questions arise: (1) Which linguistic phenomena do commenters recurrently identify as difficult or manageable? (2) Which overarching belief and attitude patterns emerge, and what role do cross-linguistic comparison, learning conditions, and evaluation play in them? (3) How do commenters negotiate the tension between perceived difficulty and the belief that the language is manageable through one’s own effort? In this way, the study connects the structural, the learning-psychological, and the discourse-analytic perspectives on the perceived difficulty of German.

## 3 Method

### 3.1 Research design

The study follows a qualitative research design and aims to reconstruct, from a learning-psychology perspective, which beliefs, attitudes, and coping expectations Turkish-speaking learners express with respect to the difficulty of the German language. Accordingly, the category system captures not primarily linguistic structures as such, but the subjective evaluations, comparisons, and self-efficacy perceptions in which these are embedded. The data consist of user-generated comments on YouTube videos. Such comments constitute an extensive textual space of computer-mediated communication that allows authentic, largely uncensored insights into attitudes and metalinguistic discourses and that, compared with direct surveys, is less subject to the tendency toward socially desirable responding (Benson, 2015; Chowchong, 2021). The analysis is carried out by means of qualitative thematic analysis following Braun and Clarke (2006), which is suited to identifying recurrent patterns of meaning in extensive text corpora and is well established in the analysis of YouTube comments (Liew and Hassan, 2021). Since language-related attitudes are not directly observable but must be inferred from verbal statements (Garrett, 2010), such comments are particularly well suited as a data basis for reconstructing the underlying beliefs and attitudes.

### 3.2 Data collection and corpus construction

Data collection took place on the YouTube platform. The selection of videos followed purposive sampling: using predefined, documented search terms (“almanca,” “almanca zor mu,” “almanca zorluk,” and “almanca kolay”), Turkish-language videos dealing thematically with the difficulty or the learning of the German language were identified. Videos were included whose comment section was active and publicly accessible and contained a sufficient number of thematically relevant comments. The selection followed the logic of purposive sampling rather than statistical representativeness. The aim was not to assemble a representative sample of all videos on the topic, but to identify a small number of videos whose comment sections were particularly rich in metalinguistic discourse about the difficulty of German. From the videos returned by the search terms, those were retained that met three criteria: a thematic focus on the difficulty or the learning of German, an active and publicly accessible comment section, and a substantial volume of thematically relevant comments that allowed for an in-depth qualitative analysis of the emerging patterns. Videos with disabled comments, with very few relevant comments, or with a purely instructional focus without reference to the perception of difficulty were excluded. The application of these criteria yielded four videos whose comment sections together provided a corpus of sufficient size and thematic density for the qualitative analysis. The concentration on a small number of comment-rich videos is characteristic of qualitative design, in which depth of analysis takes precedence over breadth.

The comments were collected using the browser-based research tool YouTube Data Tools (Rieder, 2015), which is well established in digital methods research and builds on the official endpoints of the YouTube Data API v3 (Rieder et al., 2018). The videos were identified via the video search module, which queries the API’s search endpoint; the comments of the selected videos were then exported in structured form as a table, together with their metadata. Already at the point of export, the comments were pseudonymized, so that no real names were carried over into the data set. Data collection took place in June 2026. The final analytic corpus comprises four Turkish-language videos with a total of 3,884 comments (see Table 1).

**Table 1:** Overview of the analyzed corpus.

Video-ID	Total comments	Of which coded as relevant
7N2R44W1axg	2,800	1,567
ZMDciqFjKog	622	325
RtWbz4E6U8o	266	120
Ikfel_ldIT0	196	73
Total	3,884	2,085

Four Turkish-language YouTube videos on the difficulty of the German language. “Coded as relevant” denotes comments that contained at least one analyzable statement on the perception of difficulty; non-pertinent contributions were excluded from the content analysis.

A conceptual clarification concerning the commenters is necessary. The corpus does not permit verification of whether each commenter is currently learning German; commenters may include active learners, former learners, native speakers, and other individuals engaging with the topic. The analysis therefore does not treat the commenters as a verified population of German learners. Rather, it examines the discourse about the difficulty of German as it is articulated by those who participate in it, on the assumption that this discourse is shaped predominantly, though not exclusively, by learners and by learning-related experience. Where the term “learners” is used in the following, it refers to this discourse community rather than to a verified group of enrolled learners.

### 3.3 Data preparation

The exported comments were transferred into a uniform table structure in which each comment forms its own row and is linked to the corresponding video identifier, the publication date, the number of likes, and the information on whether it is an initial comment or a reply. The authors were consistently replaced by pseudonyms. To presort the extensive material, a keyword-based pre-filtering was employed that flags comments containing thematically pertinent terms; this pre-filtering served solely to make the material accessible and did not replace the substantive reading and coding.

### 3.4 Analytic procedure

Prior to the thematic analysis proper, a relevance screening of the material was carried out. Each comment was examined for whether it contained at least one analyzable statement on the perception of the difficulty or on the learning of German. Of the 3,884 comments, 2,085 were thus classified as relevant and passed on to the content analysis, while non-pertinent contributions were excluded.

The subsequent analysis of the relevant comments followed the six phases of thematic analysis according to Braun and Clarke (2006). In the first phase, the researcher became familiar with the material through repeated reading of the comments. The analysis followed a hybrid approach. In the second phase, initial codes were generated inductively and close to the material; the predefined search terms served only to identify the videos and to preselect potentially relevant comments, not as coding categories. Multiple coding was permitted when a comment addressed several aspects. This open coding yielded 125 initial codes. In the third phase, these codes were reviewed for overlap and redundancy and consolidated into 80 codes; codes with identical or largely overlapping meaning were merged, and codes with very few instances were subsumed under broader ones, with each decision recorded in the codebook. The 80 codes were then grouped into eight main categories. This second level was formed inductively from the coded material but was structured with reference to the theoretical framework, in that categories were differentiated into four language-level categories (Phonetics/Phonology, Morphosyntax, Lexis, and Literacy & Skills) and four meta-level categories (Difficulty evaluation, Learning & Acquisition, Cross-linguistic comparison& Multilingualism, and Attitudes, Social & Meta). This division reflects the learning-psychology research interest of the study: while the language-level categories capture the structural phenomena on which perceived difficulty is fixed, the meta-level categories represent the psychologically central constructs, namely difficulty appraisals, learning- and acquisition-related beliefs including self-efficacy, and comparative and social attitudes. In the fourth phase, these themes were reviewed by relating them back to the coded comments and the overall data set; in the course of this review, comments assigned to unrelated themes were excluded or recoded. In the fifth phase, the main categories were defined, named, and substantiated with anchor examples from the corpus; the complete category system with definitions and anchor examples can be found in Supplementary Tables S1, S2. The sixth phase consisted of writing up the results in the present report. It should be emphasized that the data capture expressed beliefs and appraisals, not measured psychological constructs. Comments were the analysis relates these expressions to constructs such as self-efficacy, this is intended as a theoretically motivated interpretation of the belief patterns, not as a claim about measured self-efficacy.

Because all coding was carried out by a single researcher, several procedures were used to enhance the credibility of the analysis. The author is a native speaker of Turkish and a teacher educator in German as a foreign language; this background enabled the interpretation of idiomatic, colloquial, and ironic comments, but it also implies a professional perspective on leaning difficulty that was consciously reflected during coding. Coding decisions were documented in a codebook with explicit definitions and anchor examples, and a coding checklist was used to apply the definitions consistently across the corpus. The material was coded in several passes: after the initial coding, all comments were re-read against the consolidated codebook and recoded where necessary, so that early and late coding decisions were aligned. No independent second was conducted; this limitation is addressed in Section 3.6.

In response to the growing relevance of generative AI for language learning, the coded material was additionally examined in a targeted *post-hoc* analysis for references to AI tools, translation software, and language-learning applications. This dimension was not part of the original category system; the corresponding comments were identified through a systematic keyword-based search of the full corpus and are reported separately as an exploratory observation.

### 3.5 Research ethics

The studies involving human participants were reviewed and approved by the Scientific Research and Publication Ethics Committee of Nevşehir Hacı Bektaş Veli University (meeting no. 11, decision no. 2026.11.17, dated July 23, 2026). Because the study constitutes a retrospective analysis of pre-existing, publicly accessible data that did not involve any interaction or intervention with human participants, ethical clearance was obtained during preparation of the study for publication; the committee confirmed that the study raises no ethical objection. Written informed consent for participation was not required for this study in accordance with the national legislation and the institutional requirements, as the study is based exclusively on publicly accessible, pseudonymized data.

### 3.6 Quality criteria and limitations

The traceability of the analysis is ensured through the transparent documentation of the search strategy, the inclusion and exclusion criteria, and the complete category system with definitions and anchor examples. At the same time, several limitations must be noted. First, the data rest on self-selected, publicly expressed comments, so that particularly engaged or pointed voices are likely to be overrepresented, and the findings are not representative, in the statistical sense, of all Turkish-speaking learners of German. Second, the analysis captures expressed perceptions, not the actual learning behavior or the objective language proficiency of the commenters. Third, the coding was carried out by a single person; an independent second coding to determine intercoder agreement was not conducted, which must be taken into account when interpreting the results. Because the corpus rests on a small number of purposively selected videos, the findings may be shaped by the specific topics, audiences, and comment dynamics of these videos. The results are therefore not statistically generalizable; in line with the logic of qualitative research, their value lies in analytical transferability, that is, in the extent to which the reconstructed pattern can inform the understanding of similar discourse in comparable settings, rather than in representativeness.

Fourth, corpus construction is bound to the conditions of the platform and the data collection tool. Since the video selection was made via the search function of the YouTube Data API, it depends on localization, platform logic, and the time of retrieval, and it does not exactly mirror the search experience of individual users; which videos and comments are findable is thus co-shaped by the platform (Rieder et al., 2018). The collected corpus therefore represents a snapshot. Moreover, since videos and comments can be subsequently altered or deleted, the time of collection (June 2026) was documented in order to ensure traceability.

## 4 Results

In what follows, the results of the thematic analysis are presented. The presentation follows the three research questions: first, the overall distribution of the eight main categories is considered; subsequently, the substantive emphases are differentiated at the level of the subcategories; and finally, paraphrased anchor examples illustrate the substantive content of the statements. The basis is the 2,085 comments coded as relevant; since multiple coding was permitted, the percentages do not sum to 100%.

Table 2 shows a clear predominance of the meta-level over the language-level categories. Most frequently, the commenters addressed cross-linguistic comparison and multilingualism (*n* = 1,083; 51.9%) as well as aspects of learning and acquisition (*n* = 1,047; 50.2%), followed by explicit difficulty evaluation (*n* = 666; 31.9%) and attitude-related and social attributions (*n* = 454; 21.8%). The categories referring concretely to linguistic structures, by contrast, occurred less frequently, with morphosyntax (*n* = 301; 14.4%) taking the largest share, followed by lexis (*n* = 180; 8.6%), literacy and skills (*n* = 116; 5.6%), and phonetics/phonology (*n* = 87; 4.2%). This distribution pattern indicates that the perceived difficulty of the German language is negotiated primarily through comparative, learning-related, and evaluative references, and only secondarily through individual linguistic phenomena. RQ2 is thereby already answered in its basic outline: the interpretive patterns of difficulty are predominantly relational and biographical in nature, not primarily structural-linguistic.

**Table 2:** Frequency of the main categories in the relevant comments (*N* = 2,085).

Code	Main category	*n*	%
O7	Cross-linguistic comparison & multilingualism	1,083	51.9
O6	Learning & acquisition	1,047	50.2
O5	Difficulty evaluation	666	31.9
O8	Attitudes, social & meta	454	21.8
O2	Morphosyntax (articles/gender, case, grammar)	301	14.4
O3	Lexis (vocabulary, compounds)	180	8.6
O1	Phonetics/phonology (pronunciation, sound)	87	4.2
O4	Literacy & skills	116	5.6

*n* = number of relevant comments with at least one code of the main category. Since a comment can be assigned to several main categories, the sum of the n values exceeds *N*.

With regard to the linguistic phenomena (RQ1), a clear profile emerges within the structure-related categories, which are rarer to begin with: morphosyntax dominates, while the remaining language levels occur in a subordinate position. The differentiation at the level of the subcategories in Table 3 makes these emphases visible.

**Table 3:** Selected subcategories per main category (most frequent codes).

Main cat.	Selected subcategory (code)	*n*
O7	Comparison with Turkish	636
O7	Turkish is portrayed as difficult	252
O7	Other language groups learn German more easily	240
O6	Personal learning experience	385
O6	Willingness to learn determines success	274
O6	School/course/instruction as a learning setting	223
O5	German is portrayed as difficult	384
O5	German is relativized / not difficult	284
O5	German is difficult but learnable	195
O2	Grammar and sentence structure as a difficulty	127
O2	Articles/gender as a difficulty	67
O3	Vocabulary, individual words, or compounds	172
O8	Social group criticism and stereotyping	173
O8	Knowledge of German as a utilitarian language	86
O4	Reading, writing, or orthography	93
O1	Pronunciation/guttural sounds	48

*n* = frequency of the code in relevant comments. For each main category, the most frequent individual codes are shown; related codes were not aggregated. The complete code list can be found in Supplementary Tables S1, S2.

For the sake of clarity, only the most frequent codes are reproduced for each main category. Within the main category cross-linguistic comparison and multilingualism, the largest share falls on the reference to Turkish (*n* = 636), frequently combined with the observation that members of other language groups learn German more easily (*n* = 240). The main category learning and acquisition is borne above all by the recourse to personal learning experiences (*n* = 385) and by the conviction that the willingness to learn determines success (*n* = 274). Within morphosyntax, it is in particular grammar and sentence structure (*n* = 127) as well as articles and gender (*n* = 67) that are named as difficulty-relevant. The subcategories make clear that behind the quantitatively dominant meta-level main categories lie differentiated and partly opposing interpretations.

The main category difficulty evaluation deserves particular attention, as the tension central to RQ3 is reflected within it. The emphatic portrayal of German as difficult (*n* = 384) is matched by relativizing statements (*n* = 284), and the mediating position “difficult but learnable” emerges as a distinct pattern (*n* = 195). Difficulty is thus framed in the comments not consistently as an insurmountable barrier, but in many cases as a manageable challenge that can be overcome through effort.

While the frequency tables represent the quantitative distribution of the categories, the paraphrased anchor examples in Table 4 serve to make the substantive content of the statements comprehensible and to substantiate the category construction with the material. To protect the privacy of the commenters and to prevent re-identification through search engines, these examples are reported as content-preserving paraphrases rather than verbatim quotations.

**Table 4:** Paraphrased anchor examples per main category.

Main category	Anchor example (paraphrased)
O1 Phonetics/phonology	A commenter finds German pronunciation very enjoyable, describing certain words as satisfying to articulate fully.
O2 Morphosyntax	A commenter calls German difficult and notes that even many native Germans cannot use the articles correctly.
O3 Lexis	A commenter points to an ambiguous German word with two opposite meanings and adds that even Germans do not know the article of many words.
O4 Literacy & skills	A commenter reports encountering many foreigners who consider German easy, citing its alphabet, writing, and reading.
O5 Difficulty evaluation	A commenter asserts that anyone who claims German is not difficult clearly does not know the language.
O5 Difficulty evaluation (ambivalent)	A commenter stresses that learning German requires great persistence precisely because it is very difficult.
O6 Learning & acquisition	A commenter recounts arriving in Germany for university without any German and describes progress made after a several-month language course.
O7 Cross-linguistic comparison & multilingualism	A commenter advises that those who know English learn German through English-language materials, as this makes it easier.
O8 Attitudes, social & meta	A commenter doubts that those not born and raised in Germany ever fully master the language, even after decades of residence.

For each main category, a representative anchor example is given. To protect the privacy of the commenters and to prevent re-identification through search engines, the examples are reported as content-preserving paraphrases rather than verbatim quotations. For O5, two examples are given in order to represent the central tension between the attribution of difficulty and its framing as manageable.

The anchor examples make clear that the engagement with the German language is in many cases embedded in a biographical and comparative horizon of experience. The two examples on difficulty evaluation directly illustrate the tension central to RQ3: while the first categorically asserts the difficulty of German, the second combines the acknowledgment of difficulty with the call for persistence, thereby framing it as surmountable.

Beyond the established coping resources captured by the category system, a targeted *post-hoc* examination revealed a small but notable set of comments (*n* = 7), occurring exclusively in the most recent data from 2025 and 2026, that invoked AI tools as a means of coping with the difficulty of German. Learners referred, for instance, to AI programs for handling official written correspondence, recommended asking an AI system whether German or Turkish is the more difficult language, or described using a chatbot to check and correct their own written German. Although marginal in frequency, these comments are noteworthy because they appear only in the newest discourse and frame AI not as an object of difficulty but as a resource that relativizes it: the availability of AI support is presented as a reason not to fear the language’s demands. This pattern is exploratory and should not be overstated given its low frequency, but it points to an emerging shift in how coping with perceived difficulty is negotiated.

## 5 Discussion

The aim of the present study was to reconstruct, from a learning-psychology perspective, which beliefs, attitudes, and coping expectations Turkish-speaking learners express with respect to the difficulty of the German language. The results paint a picture in which this difficulty is negotiated less as a property of the language system itself than as something relational and biographical, that is, as a subjective appraisal embedded in beliefs, comparisons, and coping expectations. In what follows, the findings are situated along the three research questions and related to the psychological constructs of learner beliefs, language attitudes, and self-efficacy.

### 5.1 Beliefs about specific language difficulties (RQ1)

With regard to the first research question, it becomes apparent that, although the concrete language-level categories occurred less frequently than the meta-level ones, they form a clear and theoretically expectable profile within that level. Morphosyntax dominated (14.4%), and within it, it was above all grammar and sentence structure as well as articles and gender that were named as difficulty-relevant. This finding converges with the contrastive description of the language pair: since Turkish lacks grammatical gender and an article system, precisely these phenomena have no equivalent in the first language and they are the ones the commenters most frequently single out (Ceylan, 2018; cf. Balcı, 2009). The named difficulties in the area of speaking and comprehension as well as vocabulary can likewise be related to the described typological divergences (Köppel, 2017; Kalkavan, 2012). This convergence should not be read as evidence that these phenomena are objectively more difficult or produce more errors; the present data document which structures commenters perceive and talk about as difficult, not how difficult these structures are in terms of acquisition. Perceptions of this king may also be shaped by widely circulating public views of German as a hard language (Krüsemann and Graham, 2024). What the data show is that the commenters name these phenomena not in detached, technical terms but as concretely experienced stumbling blocks, so that the structural contrasts described in the contrastive literature reappear as objects of learner-side perception; it is this discursive level that the contrastive literature itself leaves open. These concretely named phenomena at the same time form the substantive basis on which the higher-order beliefs and evaluations considered in what follows are anchored.

### 5.2 Overarching interpretive patterns (RQ2)

The second research question concerned the overarching interpretive patterns. Here lies the central finding of the study: perceived difficulty is negotiated very predominantly through meta-level framings, above all through cross-linguistic comparison and multilingualism and through learning- and acquisition-related aspects, which together form the two most frequent main categories (Table 2) and clearly outweigh references to individual linguistic phenomena. What makes this pattern significant is not the distribution as such but what it reveals about how difficulty is constructed: the commenters do not primarily describe the German language, they position it. The comparison refers above all to Turkish, frequently combined with the observation that members of other language groups learn German more easily. Difficulty is thus framed relationally, as a property of the distance between one’s own language and German and of one’s position among other learner groups This pattern supports the basic assumption of language attitude research, according to which judgments about a language are socially embedded evaluations rather than mere descriptions of linguistic structures, and it corresponds to the significance of perceived difficulty as a distinct dimension of learner beliefs (Horwitz, 1988). That the commenters in this corpus frequently classify German as difficult in comparison with other languages is consistent with earlier findings on Turkish learners, according to which the perception of difficulty varies by language and context (Ariogul et al., 2009). The comparative framing has a double face, however: the recourse to one’s own multilingual repertoire may serve as a resource for appraising and structuring the task, whereas the recurrent claim that other groups learn German more easily introduces a social comparison that can locate the difficulty in one’s own group and thereby feed a deficit-oriented self-image. In interpreting these patterns, the distinction drawn by Garrett (2010) must be observed: a positive attitude toward the language as such is not to be equated with the attitude toward the learning process, and only the latter permits inferences about actual learning behavior. The beliefs reconstructed here therefore concern the expressed evaluation, not necessarily the intention to learn.

The prominence of the learning- and acquisition-related framing, in particular the recurrent idea that the willingness to learn determines success, points to the role that coping-related beliefs and possibly self-efficacy, may play in how difficulty is negotiated (Çobanoğulları, 2022; Krüsemann and Graham, 2024). The commenters thereby appear to shift the responsibility for success from the language onto the learning person and framing difficulty as something that can be influenced through effort. This pattern is directly visible in the material. Within the category of learning and acquisition, the conviction is among the most frequent codes: commenters stress, for instance, that studying the language requires discipline, argue that anyone who wants to can learn it, present frequent speaking without fear of mistakes as the key to progress, or locate the problem not in the language itself but in a rote and low-intensity way of studying. Such statements do not describe persistence as a measured trait, but they show that effort and determination are the terms in which the commenters themselves account for success and failure. This corresponds to Bandura’s (1997) observation that persons with a strong sense of efficacy tend to attribute setbacks to insufficient effort rather than to the difficulty of the task, an attributional pattern that is regarded as conducive to sustained engagement. The theoretical relevance of this finding lies in its location: such effort-oriented accounts are documented here not in response to a questionnaire but as spontaneous contributions to a public discourse, which suggests that the belief in the controllability of difficulty is part of how Turkish-speaking commenters routinely talk about German. Overall, the findings suggest that the engagement with German in the analyzed comments is borne more by comparative, experiential, and evaluative discourses than by a phenomenon-oriented consideration.

### 5.3 The tension between difficulty and manageability (RQ3)

The third research question concerned the relationship between perceived difficulty and coping expectation. The data show that both poles occur substantially and often jointly: the emphatic portrayal of German as difficult is matched by similarly frequent relativizing statements, and the mediating position “difficult but learnable” forms a distinct pattern. Psychologically, this ambivalence may be understood as a belief pattern in which a high difficulty appraisal coexists with confidence in one’s own ability to cope: the difficulty is acknowledged but at the same time framed as a challenge that can be managed through one’s own effort. It should be emphasized that self-efficacy was not measured in this study; the expressed confidence in coping is therefore interpreted as consistent with, but not equivalent to, a self-efficacy belief in Bandura’s (1997) sense, and may also reflect prior learning experience, general optimism, or learning attitudes. The material illustrates both sides of this pattern. On the one hand, commenters recount reaching an advanced certificate within a year despite demanding working conditions, or frame difficulty as relative and attainable through disciplined study; on the other hand, the same category contains accounts of years of learning with little sense of progress, of decades of residence in Germany without adequate command of the language, and of emotional exhausting in the face of German. The ‘difficult but learnable’ position thus emerges not as uniform optimism but as a stance formed against the background of such contrasting experiences. This regarding is consistent with Bandura’s (1997) conception of self-efficacy as a task-specific rather than global belief and with the view, established in learner belief research, that beliefs about language learning are contextually situated and subject to change rather than fixed traits (Horwitz, 1988; Courtney, 2017). Read in this light, the ‘difficult but learnable’ position resembles what mindset research describes as a growth-oriented belief about language learning, in which difficulty is acknowledged but treated as responsive to effort (Lou and Noels, 2017; Kim, 2024). This pattern connects directly to the assumption that a difficulty interpreted as a challenge does not necessarily demotivate but is compatible with sustained learning engagement, provided that learners believe in their own efficacy (Çobanoğulları, 2022; Krüsemann and Graham, 2024). The coexistence of attribution of difficulty and confidence in coping thus represents an original contribution of the present study, one that goes beyond the mere observation that German is regarded as difficult and that makes visible how perceived difficulty and coping-related beliefs are jointly negotiated in informal digital discourse. Theoretically, this pattern can be doubly grounded. In terms of Bandura’s (1997) framework, it is consistent with high task-specific self-efficacy: persons who trust in their capability to cope tend to perceive difficult tasks as challenges to be mastered rather than as threats to be avoided. The appraisal model of Lazarus and Folkman (1984) further allows this interplay to be captured more precisely. Applying it to language learning, the classification of German as difficult corresponds to a primary appraisal that clarifies what is at stake, while the assessment of one’s own coping options corresponds to a secondary appraisal that clarifies what can be done. Since, according to Lazarus and Folkman (1984), both appraisals shape the emotional reaction not in isolation but interactively, the ambivalence “difficult but learnable” can be understood as the result of their interplay: a demand that is difficult but assessed as controllable is appraised as a challenge rather than as a threat (challenge appraisal). In this reading, Bandura’s self-efficacy concept and the appraisal model mesh: the belief in manageability may defuse the perceived difficulty and reframe it into as a challenge that supports sustained learning engagement.

The L2 Motivation Self System (Dörnyei, 2009) adds a complementary perspective on why this belief pattern may matter motivationally. In this model, motivation arises from the effort to reduce the gap between the actual self and an ideal L2 self, provided that this ideal self is perceived as plausible and attainable; a second self-guide, the ought-to L2 self, captures externally imposed expectations, and the L2 learning experience captures situated evaluations of the learning process itself. All three components have counterparts in the material. The instrumental motives documented for the Turkish context (Section 2.2) resemble an ought-to orientation, the frequent recourse to personal learning experience corresponds to the experiential component, and the “difficult but learnable position can be read as a statement about the plausibility of an ideal L2 self: the language is demanding, but a competent future self remains within reach through effort. Recent studies support this. Reading by showing that growth-oriented beliefs about language learning relate to engagement primarily via the ideal L2 self and via perseverance of effort (Sadoughi et al., 2023; Kırmızı et al., 2023). Conversely, the recurrent comparison in which other groups are said to learn German more easily may weaken the plausibility of such a self-image and thus its motivating force. These connections remain interpretive, since the self-guides were not assessed; they indicate, however, that the appraisal of difficulty as manageable and the motivational plausibility of a future self are two sides of the same belief pattern.

Although the central finding highlights that learners frame German as “difficult but learnable”, an exploratory and highly preliminary observation in the data points to a further dimension. Specifically, a very small fraction of the analyzed YouTube comments (*n* = 7), exclusively from 2025 and 2026, mention generative artificial intelligence as a means to manage this difficulty. While these data only capture expressed references to AI rather than the measured effects of its actual use, recent empirical evidence indicates that using AI can increase learners’ self-efficacy in understanding grammar and communicating (Kittredge et al., 2025). Furthermore, engaging with these digital tools is associated with reduced language anxiety and increased motivation (Li et al., 2025), particularly when AI use satisfies learners’ basic psychological needs for autonomy and competence (Guo and Zheng, 2026). At the same time, invoking AI as a resource is not the same as deriving efficacy from it: in the Turkish context, the efficacy-building effect of self-regulated AI use has been shown to depend on the user’s critical thinking (Başaran, 2026), so that the comments analyzed here document an appraisal of AI as helpful, not its actual contribution to coping. From the appraisal perspective adopted in this study, in line with Lazarus and Folkman (1984), the commenters’ mentioning of AI tools may be tentatively interpreted as an emerging form of secondary appraisal, in which learners identify technological assistance as a possible coping resource for dealing with perceived difficulty. Given the very small number of cases, this observation should be regarded as exploratory only; as generative AI becomes more widely used, further research should systematically investigate how learners’ evolving beliefs about AI interact with their perceived language difficulty and self-efficacy.

## 6 Implications and suggestions

At the theoretical level, the findings speak most directly to the psychology of learner beliefs and self-efficacy. They indicate that the perceived difficulty of a demanding learning task should be modeled not in isolation but in interplay with self-efficacy and coping expectation. The coexistence of a high attribution of difficulty with confidence in coping suggests that models of difficulty perception should be extended by an appraisal-theoretical component that incorporates the secondary appraisal of one’s own resources (Lazarus and Folkman, 1984). This has implications for how motivation is understood: the data suggest that a high perception of difficulty does not necessarily depress motivation, but may be reframed as a manageable challenge when learners hold strong efficacy beliefs, consistent with Bandura's (1997) account of domain-specific self-efficacy and with Dörnyei's (2009) motivational self-system, in which an aspired self-image energizes effort only when it is perceived as attainable. Beliefs about learning, in this reading, may function as an appraisal lens that mediates between a task perceived as demanding and sustained motivational engagement.

A more applied set of implications follows secondarily from these psychological insights. Because the findings concern expressed beliefs rather than testes interventions, the following recommendations are formulated as hypotheses for practice rather than as validated measures. Three recommendations can be derived from the three central patterns.

First, the pattern “difficult but learnable” and the prominence of effort-oriented accounts suggest that instruction should neither deny the difficulty of German nor leave learners alone with it, but make manageability experienceable. Concretely, teachers could acknowledge openly that features such as gender, articles and case are demanding for Turkish-speaking learners and at the same time structure learning in visible stages with milestones at which progress is documented and reviewed, for instance through portfolio entries or short can-do checks tied to the curriculum. Feedback that foregrounds effort and strategy use than ability is consistent with the attributional pattern found in the comments and, in line with Bandura’s (1997) account of mastery experiences, may strengthen the belief that difficulty responds to effort.

Second, the dominance of comparative framings indicates that learners already reason contrastively; instruction can use this rather than ignore it. Structural contrasts between Turkish and German (Section 2.4) could be addressed explicitly as expected and normal hurdles rather than as signs of individual or group deficiency and where learners know English, the typologically closer language can be used systematically as a bridge for vocabulary and sentence structure, as proposed in tertiary language didactics (İşigüzel,2025). Explicitly reframing the recurrent belief that other groups learn German more easily as a matter of typological distance rather than of ability may counter deficit-oriented self-images.

Third, the fact that beliefs about German are formed and negotiated in informal digital spaces suggests that such spaces can be brought into the classroom as objects of reflection. Authentic comment discourse in anonymized or paraphrased form, could be used as material for learners to identify, compare and question the beliefs about difficulty and learnability they encounter online and hold themselves, an approach for which the use of YouTube comments as authentic material has already been proposed (Cotgrove, 2023). Making beliefs explicit in this way is a precondition for addressing them, and it connects the classroom to the discourses in which learners actually seek orientation.

## 7 Conclusion

The present study has reconstructed, from a learning-psychology perspective, which beliefs, attitudes, and coping expectations Turkish-speaking commenters associate with the difficulty of the German language, drawing on the informal discourse in YouTube comments. Three findings stand out. First, the concrete features named as difficult concentrate on morphosyntax, above all articles, gender and sentence structure and thus on precisely those areas in which Turkish offers no equivalent. Second, difficulty is negotiated predominantly in relational and biographical terms, through comparison with Turkish and other languages, through accounts of learning experience and through evaluations of the learning process and only secondarily through individual linguistic phenomena. Third, the acknowledgment of difficulty coexists with the conviction that German can be learned through effort. This “difficult but learnable” position is the characteristic pattern of the corpus; we read it as a belief pattern consistent with an interplay of perceived difficulty and coping-related beliefs, not as evidence of measured self-efficacy.

The findings are to be read in light of their limitations. The data stem from publicly visible, self-selected comments, so that engaged or pointed voices are likely to be overrepresented; the analysis captures expressed perceptions, not actual learning behavior; the coding was carried out by a single person without independent second coding; and the corpus obtained through the platform search represents a time-bound snapshot. Future research could secure the patterns reconstructed here through independent multiple coding, extend them through comparison with other platforms or language groups, and connect the expressed beliefs with standardized measures of self-efficacy and with data on actual learning behavior. Despite these limitations, the study demonstrates the value of user-generated digital data for understanding how learners interpret a language and its difficulty, and it suggests that perceived difficulty becomes psychologically intelligible in interplay with the belief in its manageability.

Finally, the emergence of AI-related coping in the most recent comments points to a promising direction for future research. As generative AI tools become a routine part of language learning, investigating how learners’ beliefs about AI interact with their perceived difficulty and self-efficacy, and whether AI reshapes the appraisal of a language as difficult but manageable, appears both timely and necessary.

## Data Availability

The original contributions presented in the study are included in the article/Supplementary material, further inquiries can be directed to the corresponding author.

## Supplementary material

SUPPLEMENTARTY TABLE S1 Codebook with code definitions and paraphrased anchor examples.

SUPPLEMENTARY TABLE S2 Frequencies of all codes in the relevant comments.
