# Supplementary material for: Between difficulty and manageability: beliefs about the learnability of German in Turkish-language YouTube comments
Source: Front Psychol. 2026 Oct 8;17:1926209. doi: 10.3389/fpsyg.2026.1926209 (PMC13687042; doi:10.3389/fpsyg.2026.1926209)
Supplement: Supplementary file 1 [file Table_1.DOCX]

**Supplementary Table S1. Codebook with code definitions and paraphrased anchor examples.**

*All content codes of the thematic analysis, organized by the eight main categories. Each code is given with its definition and one anchor example. To protect the privacy of the commenters and to prevent re-identification through search engines, anchor examples are reported as content-preserving paraphrases rather than verbatim quotations.*

**O1 · Phonetics & Phonology**

| **Code** | **Definition** | **Anchor example (paraphrased)** |
| --- | --- | --- |
| **Pronunciation, accent, or articulation** | Statements addressing the pronunciation, accent, stress, or individual sounds of German. | A commenter describes German as the most difficult language specifically with regard to pronunciation. |
| **Pronunciation/guttural sounds problematized** | Statements problematizing specific sounds of German, especially guttural and throat sounds. | A commenter links good pronunciation and grammar to early, immersive acquisition in the country, implying that pronunciation is otherwise hard to master. |
| **German sounds coarse/harsh or unpleasant** | Statements evaluating the sound of German as coarse, harsh, or unpleasant. | A commenter perceives the ch and similar German sounds as coarse. |
| **German sounds beautiful/pleasant** | Statements evaluating the sound of German as beautiful or pleasant. | A commenter finds German pronunciation very enjoyable and describes certain words as satisfying to articulate fully. |
| **Pronunciation interference** | Statements attributing pronunciation difficulties to interference with the first language. | A commenter reports having vocabulary knowledge but being unable to speak, attributing the difficulty specifically to pronunciation. |

**O2 · Morphosyntax**

| **Code** | **Definition** | **Anchor example (paraphrased)** |
| --- | --- | --- |
| **Grammar and sentence structure as a difficulty** | Statements naming grammar and sentence structure of German as a difficulty. | A commenter reports having learned German grammar within months and then expanding it through everyday social contact. |
| **Articles/gender as a difficulty** | Statements naming articles or grammatical gender as a difficulty. | A commenter notes that even native Germans are unsure of the article of many words. |
| **Grammar/regularity/wealth of detail at the center** | Statements placing the regularity and wealth of grammatical detail of German at the center. | A commenter characterizes German as rule-governed and emphasizes that studying it requires discipline. |
| **Articles/gender marked as a problem** | Statements explicitly marking articles or gender as a problem. | A commenter points to an ambiguous verb whose form remains identical despite opposite meanings, treating it as an oddity of the grammar. |
| **Case/declension as a difficulty** | Statements naming case or declension as a difficulty. | A commenter frames case and inflectional suffixes as a source of frequent grammatical errors, drawing a comparison with Turkish. |
| **German as logical or rule-governed** | Statements portraying German as logical or rule-governed. | A commenter questions the claim that a language can be mathematical, arguing that any language is logical if one looks for logic. |
| **Case/declension/dative/accusative impede learning** | Statements in which case, declension, dative, or accusative impede learning. | A commenter judges German to be considerably easier than English, downplaying case and declension as obstacles. |
| **German or articles are difficult for beginners** | Statements portraying German or its articles as difficult for beginners. | A commenter reacts to a condescending tone by insisting that German is quite difficult for those who do not know it. |
| **Concrete grammar topics** | Statements that ask about or name concrete grammar topics. | A commenter asks how one is supposed to memorize German prepositions, listing several as examples of the difficulty. |
| **Grammatical gender read as biological gender or sexism** | Statements interpreting grammatical gender as biological gender or as sexism. | A commenter interprets grammatical gender as reinforcing sexism. |
| **Grammar confusing** | Statements experiencing grammar as confusing or complicated. | A commenter names grammar as making German one of the most difficult languages in the world. |
| **Articles interpreted as without clear logic** | Statements interpreting the articles as following no clear logic. | A commenter demands a logical explanation for why a given gender is assigned to a given object, concluding that the articles follow no logic. |
| **Prepositions and case** | Statements naming prepositions and case government as difficult. | A commenter reports struggling with verbs and their government, unsure which take the accusative and which the dative. |
| **Articles are difficult for learners** | Statements portraying the articles as difficult for learners. | A commenter rejects the claim that the der-die-das distinction is easy, calling the articles the hardest aspect of German. |
| **Comparison with other gendered languages** | Statements comparing German article/gender with other gendered languages. | A commenter compares German gender marking with another gendered language, citing parallel forms as illustration. |
| **Logic of articles demanded** | Statements demanding a logic behind the articles. | A commenter asserts that there is no underlying sense to how German articles are assigned. |
| **Article assignment interpreted as unsystematic and historically grown** | Statements interpreting article assignment as unsystematic, historically grown, or to be memorized rather than derived. | A commenter states that German provides no rule determining which article a word takes, framing article assignment as unsystematic. |
| **Article problem** | Statements naming the der-die-das article distinction as the main problem. | A commenter identifies the der-die-das article distinction as the main grammatical difference from English and the central problem. |
| **Prepositions/conjunctions/grammatical subtleties impede** | Statements in which prepositions, conjunctions, or grammatical subtleties impede learning. | A commenter describes German prepositions and conjunctions as maddeningly difficult. |
| **Turkish source language without gender makes acquisition harder** | Statements in which the absence of gender in Turkish makes German acquisition harder. | A commenter notes that grammatical gender exists in many other languages, situating the German article system within a broader cross-linguistic pattern. |

**O3 · Lexis**

| **Code** | **Definition** | **Anchor example (paraphrased)** |
| --- | --- | --- |
| **Vocabulary, individual words, or compounds** | Statements addressing vocabulary, the amount of words, individual words, or compound words of German. | A commenter recalls arriving in Germany for university without knowing a single word of German. |
| **Long words/compounds stand out as difficult or curious** | Statements in which long words or compounds stand out as difficult or curious. | A commenter reacts to German compounds by objecting that separate words have simply been strung together. |
| **Lack of vocabulary** | Statements naming missing or insufficient vocabulary as a problem that impedes expression. | A commenter, just starting out, reports knowing only the German words for yes and no. |

**O4 · Literacy & Skills**

| **Code** | **Definition** | **Anchor example (paraphrased)** |
| --- | --- | --- |
| **Reading, writing, or orthography** | Statements addressing reading, writing, or spelling of German. | A commenter mentions coming to Germany for university study, in the context of reading and writing demands. |
| **Speaking difficult** | Statements naming speaking or oral production as particularly difficult. | A commenter claims to have learned to form correct German sentences within days, contrasting this with years of unsuccessful English learning. |
| **Practical speaking central** | Statements presenting practical speaking as central to learning success. | A commenter urges frequent speaking with others without fear of making mistakes as the key to progress. |
| **Listening difficult** | Statements naming listening comprehension as difficult. | A commenter observes that although the grammar rules may be learned, listening comprehension already feels difficult at the A1 level. |
| **Writing difficult** | Statements naming writing as difficult. | A commenter reports being able to translate a sentence yet feeling unable to produce such writing independently. |
| **Translation/production problem** | Statements presenting translation or language production as a problem. | A commenter expresses frustration and emotional exhaustion at the constant demand to translate. |
| **Reading difficult** | Statements naming reading as difficult. | A commenter argues that memorizing is the easy part and that listening comprehension is the truly difficult skill. |

**O5 · Difficulty evaluation**

| **Code** | **Definition** | **Anchor example (paraphrased)** |
| --- | --- | --- |
| **German is portrayed as difficult** | Statements that emphatically or sweepingly evaluate German as a difficult language, including intensifying forms. | A commenter asserts that anyone who denies German is difficult simply does not know the language. |
| **German is relativized or called not difficult** | Statements that relativize the difficulty of German, portray it as exaggerated, or evaluate it as not difficult, including in comparison with other languages. | A commenter dismisses claims of German's difficulty as exaggerated, questioning the competence of those featured. |
| **German is difficult but learnable/manageable** | Statements that frame German as difficult but, through effort, willingness to learn, or practice, as manageable and learnable. | A commenter dismisses the interviewees' competence and argues that anyone who genuinely wants to can learn German easily, concluding it is not difficult. |
| **Practice and environment facilitate learning** | Statements that portray practical use and the linguistic environment as reducing difficulty. | A commenter presents frequent, fearless speaking with others as a path to rapid progress within months. |
| **German portrayed as easy or quickly learnable** | Statements that explicitly portray German as easy, simple, or quickly learnable. | A commenter contrasts years of unsuccessful English learning with rapid German progress, concluding that German is far easier. |
| **Long learning duration as a difficulty** | Statements that name the long duration of the learning process as an aspect of difficulty. | A commenter reacts with dismay to being told that learning German takes as long as four years. |
| **Official/technical language complicates everyday life** | Statements that name official or technical language as an additional complication in everyday life. | A commenter raises the role of English as the dominant language of science while discussing official and technical registers. |
| **German is devalued** | Statements that evaluate German in a devaluing or hostile manner. | A commenter expresses intense hostility toward German, blaming it for personal distress. |

**O6 · Learning & Acquisition**

| **Code** | **Definition** | **Anchor example (paraphrased)** |
| --- | --- | --- |
| **Personal learning experience is invoked** | One's own learning experiences cited as evidence or justification. | A commenter recounts having learned German grammar within months and then broadening it through social interaction. |
| **Willingness to learn determines success** | Willingness to learn or motivation presented as decisive for learning success. | A commenter stresses that learning German requires great persistence precisely because it is very difficult. |
| **School, course, or instruction as a learning setting** | School, course, or instruction as the setting, condition, or object of criticism of learning. | A commenter reports obtaining a C1 certificate within a year while working demanding shifts. |
| **Upbringing, age, or early acquisition affects learning** | Age, conditions of upbringing, or early acquisition as a factor influencing learning. | A commenter attributes optimal pronunciation and grammar to early, immersive childhood acquisition in the country. |
| **Speaking, dialogue, or immersion as a learning path** | Speaking, dialogue, immersion, or residence in the country as an effective learning path. | A commenter living alone in Germany reports an intermediate level and asks for help with learning. |
| **Learning resources, media, or strategies** | Concrete learning resources, media, methods, or strategies. | A commenter asks whether there is a reliable resource for learning German. |
| **Living in Germany without German is criticized** | Criticism of living in Germany without knowledge of German. | A commenter invokes the common expectation that those living in Germany should adapt to local conditions. |
| **Motivation, compulsion, or practical utility** | Motivation, compulsion, or the practical utility of German for migration, study, or work. | A commenter expresses respect for the presenter's work and hope of being able to learn German. |
| **Discipline, diligence, and self-directed work are decisive** | Diligence, discipline, regularity, or self-directed work presented as decisive. | A commenter frames difficulty as relative and argues that disciplined, determined study makes near-perfect learning possible. |
| **Exams, proficiency levels, or learning duration as a hurdle** | Exams, proficiency levels, or the long duration of learning as a hurdle in the learning process. | A commenter, at the highest proficiency level in Germany, reports that teachers describe the final exam as rarely passed on the first attempt. |
| **Residence alone does not guarantee acquisition** | Residence in the German-speaking area alone does not automatically lead to language acquisition. | A commenter with decades of residence in Germany reports still being unable to speak the language properly. |
| **Frustration, demotivation, or impulse to give up** | Frustration, hatred, demotivation, or the impulse to give up. | A commenter admits emotional exhaustion, describing German as the only language worked on to the point of tears. |
| **Learning progress, stagnation, or attrition** | Learning progress, stagnation, or the loss of acquired knowledge. | A commenter reports years of learning with little sense of progress. |
| **Method, not the language, is the problem** | Not the language but the learning method named as the actual problem. | A commenter locates the problem not in the language but in a rote, low-intensity learning method. |
| **Even native speakers or teachers do not know the rule** | Even native speakers or teachers do not reliably know the rules. | A commenter recounts a teacher's apparent lack of preparation as evidence about instruction. |
| **Language learning takes time and habituation** | Time and habituation as a prerequisite for language acquisition. | A commenter frames language learning as requiring time and habituation, likening it to something that becomes easier with continued immersion. |

**O7 · Cross-linguistic comparison & Multilingualism**

| **Code** | **Definition** | **Anchor example (paraphrased)** |
| --- | --- | --- |
| **Comparison with Turkish** | Direct comparison of German with Turkish regarding difficulty, proximity, distance, or thought structure. | A commenter disputes claims of German's difficulty and questions the Turkish competence of those featured, drawing a comparison with Turkish. |
| **Turkish is portrayed as difficult** | Turkish itself is portrayed as a difficult language. | A commenter notes that Turkish, too, is very difficult, including for Germans, framing persistence as necessary in any language. |
| **Other language groups learn German more easily** | Other language groups are credited with easier access to German. | A commenter contrasts the integration expectations placed on one group in Germany with the situation in Turkey. |
| **Mother tongue or language family determines perceived difficulty** | First language, language family, proximity, or distance presented as determining perceived difficulty. | A commenter reports rapid certification while working shifts and challenges others' command of their own first language as a precondition for learning another. |
| **Turkish is relativized as easy or self-evident** | Turkish is relativized as easy or as self-evident for native speakers. | A commenter judges Turkish to be relatively easy compared with German. |
| **Comparison with English** | Comparison of German with English, including as a contrast foil or regarding simplicity and utility. | A commenter contrasts the two languages, characterizing English as very easy and German as difficult. |
| **English knowledge or Germanic proximity facilitates German** | English knowledge or Germanic language proximity presented as facilitating the learning of German. | A commenter states that good English makes learning German easier while adding that English is not a prerequisite. |
| **Multilingual experience as justification** | One's own or family multilingualism cited as justification or evidence. | A commenter reacts with amusement to another's claim of multilingualism, listing the languages named. |
| **Language competence or multilingualism is evaluated or doubted** | The language competence of Turkish speakers or the claimed multilingualism of others is evaluated or doubted. | A commenter argues that nothing comes easily and that the will to learn must be grounded even in one's own first language. |

**O8 · Attitudes, Social & Meta**

| **Code** | **Definition** | **Anchor example (paraphrased)** |
| --- | --- | --- |
| **Social group criticism and stereotyping** | Criticism of, or stereotypical attribution to, social groups. | A commenter makes a stereotyping claim that migration to Germany is easier for one social group than for Turks. |
| **German as a utilitarian or purpose-bound language** | German is classified primarily as a utilitarian or purpose-bound language. | A commenter recounts having learned German grammar quickly and then expanding it socially, framing the language in utilitarian terms. |
| **Language as an integration or obligation expectation** | Knowledge of German is framed as an integration or obligation expectation. | A commenter frames mastery of German as an integration expectation, doubting that those not raised in Germany ever achieve it. |
| **Self-doubt, anxiety, or feeling overwhelmed as a learning emotion** | Self-doubt, anxiety, or feeling overwhelmed in connection with learning. | A commenter expresses intense distress associated with German learning. |
| **Metacomment on the video or the presenter** | Metacommunicative reference to the video, its production, or the presenter. | A commenter directs a metacomment at the presenter, accusing the video of teaching nothing and demeaning others for attention. |
| **Humor, meme, or exaggeration frames the difficulty** | The difficulty is framed through humor, meme, or exaggeration. | A commenter uses a joking question-and-answer exchange to imply, through humor, that those who dismiss German as easy do not actually know it. |
| **Explaining instead of demeaning is demanded** | A demand that learners be taught rather than demeaned; demeaning style is criticized as off-putting. | A commenter criticizes a condescending tone and demands that learners be taught rather than demeaned. |
| **Affection, interest, or positive bond with German** | Affection, interest, or a positive, also culturally appreciative bond with German. | A commenter expresses a culturally appreciative motivation, wishing to learn German in order to read a classic author in the original. |
| **Language knowledge as status or superiority** | Language knowledge is marked as status or superiority. | A commenter criticizes another's display of superiority based on claimed multilingualism. |
| **Technical or scientific metalanguage is problematized** | Technical language, scientific metalanguage, or general language questions are problematized or discussed. | A commenter problematizes the logic of grammatical gender, citing native speakers as also acknowledging its arbitrariness. |
| **German is evaluated as useless or avoidable** | German is evaluated as useless, avoidable, or not recommendable. | A commenter evaluates German as a waste of effort and recommends against learning it, citing personal regret. |
| **Social experiences with Germans influence evaluation** | Social experiences with Germans or Germany are presented as shaping the evaluation. | A commenter voices hostility toward German shaped by broader social attitudes, contrasting it with allegiance to Turkish. |
